# Optimal cerebral perfusion pressure in patients with intracerebral hemorrhage: an observational case series

**DOI:** 10.1186/cc13796

**Published:** 2014-03-25

**Authors:** Jennifer Diedler, Edgar Santos, Sven Poli, Marek Sykora

**Affiliations:** 1 Departments of Neurology and Neurosurgery, University of Tübingen, Hoppe-Seyler-Str. 3, 72076 Tübingen, Germany; 2 Department of Neurology, University of Heidelberg, Im Neuenheimer Feld 400, 69120 Heidelberg, Germany; 3 Department of Neurosurgery, University of Heidelberg, Im Neuenheimer Feld 400, 69120 Heidelberg, Germany; 4 Department of Neurology, University of Tübingen, Hoppe-Seyler-Str. 3, 72076 Tübingen, Germany

## Abstract

**Introduction:**

Current guidelines for spontaneous intracerebral hemorrhage (ICH) recommend maintaining cerebral perfusion pressure (CPP) between 50 and 70 mmHg, depending on the state of autoregulation. We continuously assessed dynamic cerebral autoregulation and the possibility of determination of an optimal CPP (CPPopt) in ICH patients. Associations between autoregulation, CPPopt and functional outcome were explored.

**Methods:**

Intracranial pressure (ICP), mean arterial pressure (MAP) and CPP were continuously recorded in 55 patients, with 38 patients included in the analysis. The pressure reactivity index (PRx) was calculated as moving correlation between MAP and ICP. CPPopt was defined as the CPP associated with the lowest PRx values. CPPopt was calculated using hourly updated of 4 hour windows. The modified Rankin Scale (mRS) was assessed at 3 months and associations between PRx, CPPopt and outcomes were explored using Pearson correlation and Fisher’s exact test. Multivariate stepwise logistic regression models were calculated including standard outcome predictors along with percentage of time with PRx >0.2 and percentage of time within the CPPopt range.

**Results:**

An overall PRx indicating impairment of pressure reactivity was found in 47% of patients (*n* = 18). The mean PRx and the time spent with a PRx > 0.2 significantly correlated with mRS at 3 months (r = 0.50, *P* = 0.002; r = 0.46, *P* = 0.004). CPPopt was calculable during 57% of the monitoring time. The median CPP was 78 mmHg, the median CPPopt 83 mmHg. Mortality was lowest in the group of patients with a CPP close to their CPPopt. However, for none of the CPPopt variables a significant association to outcome was found. The percentage of time with impaired autoregulation and hemorrhage volume were independent predictors for acceptable outcome (mRS 1 to 4) at three months.

**Conclusions:**

Failure of pressure reactivity seems common following severe ICH and is associated with unfavorable outcome. Real-time assessment of CPPopt is feasible in ICH and might provide a tool for an autoregulation-oriented CPP management. A larger trial is needed to explore if a CPPopt management results in better functional outcomes.

## Introduction

The state of cerebral autoregulation is related to functional outcome as has been shown in several studies including ischemic stroke [1], spontaneous intracerebral hemorrhage (ICH) [2,3], subarachnoid hemorrhage (SAH) [4] and traumatic brain injury (TBI) [5]. With the pressure reactvity index (PRx), Czosnyka *et al*. have introduced a method for continuous monitoring of cerebrovascular reactivity which can be used at the bedside [6]. Of importance, the PRx can be used to calculate the ‘optimal cerebral perfusion pressure’ (CPPopt) which is defined as the cerebral perfusion pressure (CPP) associated with the lowest values of PRx [5]. Studies of traumatic brain injury [5,7] and subarachnoid hemorrhage [8] have shown a relationship between outcome and the difference between the actual CPP and CPPopt. Just recently an algorithm to continuously assess CPPopt in regularly updated four-hour intervals has been published [7]. However, for spontaneous ICH there are only limited data available on dynamic autoregulation [2,3,9,10] and CPP management [11,12] and, so far, there are no data regarding autoregulation-based CPP management. However, current guidelines recommend maintaining CPP between 50 and 70 mmHg (Class IIb, Level C), depending on the state of autoregulation [13,14], conceding that these recommendations are entirely based on data from TBI patients.

Here, we (1) continuously assessed the state of cerebrovascular reactivity in patients with severe spontaneous ICH treated on a Neurocritical Care Unit (NCCU) and (2) investigated if the CPPopt concept is applicable for ICH patients. Furthermore, the association of impaired autoregulation and CPPopt to functional outcome as reported for TBI [5] and SAH [8] patients was exploratively investigated.

## Methods

### Patients

Between 2007 and 2009, 55 non-consecutive patients with spontaneous ICH admitted to the Heidelberg NCCU were prospectively included in our monitoring database. Inclusion criteria were: (1) spontaneous ICH and (2) the need for intracranial pressure (ICP) measurement. Our clinical standard protocol includes ICP measurement for all ICH patients who are analgosedated and mechanically ventilated. Furthermore, if patients have an indication for placement on an extraventricular drainage due to intraventricular extension of the hemorrhage, we usually aim to place a Raumedic NEUROVENT (RAUMEDIC AG, 95233 Helmbrechts, Germany) probe, allowing ICP measurement during simultaneous cerebrospinal fluid (CSF) drainage (see below). All patients required intubation and mechanical ventilation and were continuously sedated using midazolam, sufentanil, ketamine and/or propofol. A total of 18 patients had been included in a previous study [2]. Therapy was aimed to keep ICP <20 mmHg and CPP >60 mmHg according to current guidelines and local standards [13]. All patients were ventilated to achieve partial arterial oxygen pressures above 80 mmHg and a partial pressure of carbon dioxide in the blood (P_a_CO_2_) of 30 to 40 mmHg. ICH severity and neurological deficit on admission were assessed by the National Institutes of Health Stroke Scale (NIHSS). Hematoma volume was calculated from the first computed tomography (CT) scan using the axbxcx0.5 method [15]. The amount of intraventricular blood was estimated using the Graeb score [16]. The SAPS II (Simplified Acute Physiology Score II) on admission was extracted from the hospital patient database as a measure for overall disease severity. Outcome at three months was assessed using the modified Rankin Scale (mRS) based on a telephone interview or evaluation of the reports from the rehabilitation clinic.

The study was part of a larger neuromonitoring project. The retrospective analysis of the monitoring data was approved by the local ethics committee (Ethics Committee University of Heidelberg, S-139/2007, Amendment IV). For retrospective data analysis, the need for informed consent was waived.

### Neuromonitoring and data recording

Intracranial pressure was measured using a Raumedic NEUROVENT probe, providing a combined probe allowing ICP measurement during simultaneous CSF drainage. In six patients, ICP was measured by a standard external ventricular drain (EVD). These patients were excluded from the current analysis since ICP values are not correctly measured via an open EVD. Blood pressure was measured from the radial artery (Dräger, Medical Deutschland GmbH, Lübeck, Germany). ICP, systolic and diastolic blood pressure, mean arterial pressure (MAP), CPP and heart rate were synchronously recorded with a sampling frequency of 1 Hz using a commercially available software (ICU pilot, M Dialysis AB, Johanneshov, Sweden). The data were stored on a bedside computer.

### Assessment of pressure reactivity index (PRx)

Data files were cleaned from epochs containing incomplete data recordings. Incomplete recordings were caused by disturbed interaction between the monitoring system and the recording software or by complete disconnection of the patient from the monitoring system (for example, in-house transportations). Next, data were re-sampled to obtain one value every 10 seconds. Then, PRx was calculated as a moving linear (Pearson) correlation between 30 consecutive values (=five-minute time window) of MAP and ICP as described by Aries *et al*. [7]. For calculation of PRx only, data points fulfilling the criteria MAP between 50 and 120 mmHg, ICP >0 mmHg and CPP between 30 and 120 mmHg were included into the analysis, thereby aiming to further eliminate artifacts and outlier values, for example, due to flushing of the arterial system or shorter disconnections of one probe. According to the literature, a PRx >0.2 was defined as impaired cerebrovascular reactivity [6]. For each patient the time spent with a PRx >0.2 was calculated as the percentage of the total monitoring time. Furthermore, PRx values were averaged using Fisher-Z-Transformation to obtain an average value for each patient. All calculations were performed using Matlab (version R2007b, Mathworks, Ismaning, Germany).

### Assessment of CPPopt

**Figure 1:**
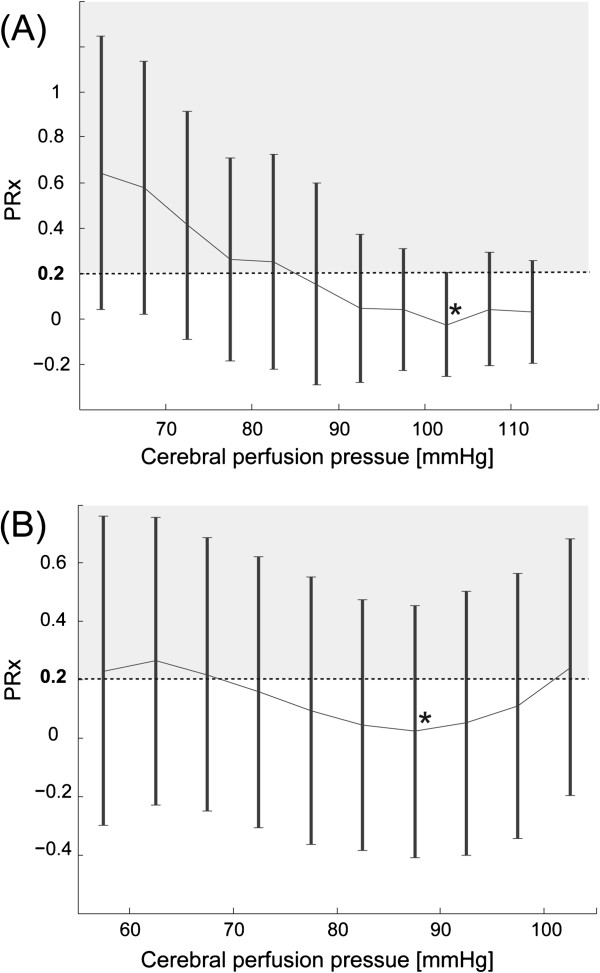
**Determination of CPPopt in two exemplary patients.** The pressure reactivity index (PRx) is plotted against cerebral perfusion pressure (CPP). PRx values >0.2 indicate impaired pressure reactivity. Patient **(A)** reaches the PRx minimum for CPP values around 103 mmHg (black star). The patient **(B)** has the optimal CPP around 87 mmHg (black star). Error bars indicate standard errors.

The optimal CPP (CPPopt) was calculated according to the algorithm published by Aries *et al*. [7]. Shortly, all recorded CPP values of each patient were divided into bins of 5 mmHg and corresponding PRx values were averaged using Fisher-Z-transformation within these groups. Fisher-Z-transformation was used because of the expected non-normal distribution of PRx values. CPPopt in general is defined as the CPP value associated with the lowest average value of PRx (see Figure 1 for an example). For continuous calculation and update of CPPopt values, the calculation was repeated every hour using a four-hour moving time window. An automatic algorithm to identify CPPopt was used as published by Aries *et al*. [7]. Furthermore, the difference between the real CPP and CPPopt was continuously calculated within the four-hour moving time window. A median value of the continuously calculated difference between the actual CPP and CPPopt was recorded for each patient and used for further analysis (Δ). The percentage of the total monitoring time during which CPPopt was calculable was recorded for each patient. In addition, the time each patient spent within (CPPopt ± 5 mmHg), below (CPP < CPPopt-5 mmHg) and above (CPP > CPPopt + 5 mmHg) the continuously updated CPPopt was calculated as the percentage of the total monitoring time. Finally, CPPopt was identified based on the entire monitoring data available for each patient as described by Steiner *et al*. [5].

### Statistical data analysis

Spearman rank correlation was used in univariate analysis to assess the relationship among PRx, time spent within, below and above the CPPopt range and outcome at three months.

In addition, patients were grouped according to their median difference between real CPP and CPPopt (three groups: median Δ ±5 mmHg within CPPopt range, median Δ >5 mmHg above CPPopt and median Δ >5 mmHg below CPPopt). The distribution of dichotomized outcome parameters (mortality, acceptable (mRS 1 to 4) and favorable (mRS 1 to 3) outcome) was compared using Fisher’s exact test.

Finally, separate forward stepwise logistic regression models were calculated to predict mortality, and favorable and acceptable outcome at three months. A stepwise model was chosen due to the explorative nature of the analysis. Due to the limited number of patients, only the known outcome predictors including age (years), initial hemorrhage volume (ml), presence of intraventricular blood in addition to percentage of time with PRx >0.2 and percentage of time within the CPPopt range were included in the model. To account for general disease severity in this special subset of ICU patients, the SAPS II score was also included in the model. There was no significant correlation between the included covariates. Statistical analysis was performed using the SPSS software package (IBM Deutschland GmbH, Ehningen, Germany).

## Results

### Patients’ characteristics and outcomes

In total, 55 patients with spontaneous ICH were prospectively monitored. Of these, four were excluded because of monitoring time <12 hours or CPP below 30 mmHg, one because of early withdrawal of care, one because of terminal stage malignoma and one because of generalized brain edema in a follow-up CCT. Additionally, the five patients with purely intraventricular hemorrhage were excluded as well as the patients who had standard EVDs without continuous ICP monitoring. All patients in whom full therapeutic measures initially were undertaken have been included in our analysis. This left 38 patients for the current analysis.

During their ICU stay, in three patients it was decided not to escalate therapeutic measures (days 7, 8, 17), two of these died. The mean age was 58 years (±SD 16, range 18 to 84 years). Median NIHSS at admission was 30.5 (IQR 20, range 7 to 39). The median SAPS II on admission was 13 (IQR 5.3, range 6 to 32).

Median hematoma size was 36 ml (IQR 50, range 3 to 144 ml), 34 patients (89.5%) had intraventricular hemorrhage extension, the median Graeb score was 6.5 (IQR 5, range 9 to 11). A total of 32 patients (84.2%) had deep hemorrhage, 5 (13.2%) lobar and 1 brainstem and cerebellar hemorrhage (2.6%). With respect to etiology, 23 patients (60.5%) had hypertensive hemorrhage, 4 had hemorrhage associated with coagulopathy (10.5%), 5 had hemorrhage of other etiologies (13.2%) and for 6 patients no etiology could be determined (15.8%).

Median total monitoring time was 78 hours (IQR 74, range 21 to 190 hours). A total of 12 (31.6%) patients underwent hematoma evacuation, 2 (5.3%) had hemicraniectomy and 1 (2.6%) had hematoma evacuation and hemicraniectomy (for this patient only data before surgery were available).

At three months, 3 patients (8.1%) attained a mRS of 2, 7 patients (18.9%) a mRS of 3, 11 patients (29.7%) a mRS of 4, 5 patients (13.5%) a mRS of 5. In-hospital mortality was 23.7% (n = 9), mortality at three months was 29.7% (n = 11). For one patient outcome at three months was not available.

### Analysis of cerebrovascular pressure reactivity

The mean PRx was suggestive for impaired pressure reactivity defined as PRx >0.2 in 47.4% (n = 18) of patients. The median time spent in PRx ranges >0.2 was 45.4% of the total monitoring time (IQR 23.0, range 15.9 to 95.1).

The mean PRx and the time spent with a PRx >0.2 were significantly correlated to mRS at three months (r = 0.50, *P* = 0.002 and r = 0.46, *P* = 0.004).

In our cohort, autoregulatory failure was not correlated to ICH severity: neither the mean total PRx nor the mean PRx on the first day of monitoring were significantly correlated to admission status assessed by NIHSS (r = −0.23, *P* = 0.159 and r = -0.22, *P* = 0.181) or hemorrhage volume (r = 0.14, *P* = 0.398 and r = 0.09, *P* = 0.605).

### Analysis of optimal CPP (CPPopt)

Figure 1 shows two exemplary patients to depict the CPPopt concept.

CPPopt was calculable during a median 57.1% of the total monitoring time (IQR 27.3, range 0 to 92.9). In a single patient, CPPopt was not calculable at all. The median of all continuously calculated CPPopt values was 83 mmHg (IQR 10, range 68 to 98). The median of all continuously calculated actual CPP values was 78 mmHg (IQR 8, range 62 to 92). The median CPPopt based on the CPP and PRx values of the entire monitoring time was 93 mmHg (IQR 12.5, range 68 to 113). The median CPP of the entire monitoring time was 79 mmHg (IQR 9, range 61 to 92). Median ICP was 10 mmHg (IQR 3.5, range 2 to 22).

Patients spent 10.5% of their total monitoring time within the continuously updated CPPopt range (IQR 10.8, range 0 to 33%). The percentage of time above the CPPopt range was 15% (IQR 13.1, range 0 to 64) and the time spent below the CPPopt range was 26.7% (IQR 23.6, range 0 to 59) in the median. The continuously calculated difference between CPP continuous CPPopt (Δ) was -5.6 mmHg (IQR 9.0, range -12.9 to 9.4) in the median.

### CPPopt and outcome

There was no linear correlation between mRS at three months and the times spent within, above or below the continuously updated CPPopt. Furthermore, functional outcome was not correlated to total monitoring time (r = -0.045, *P* = 0.791).

**Figure 2:**
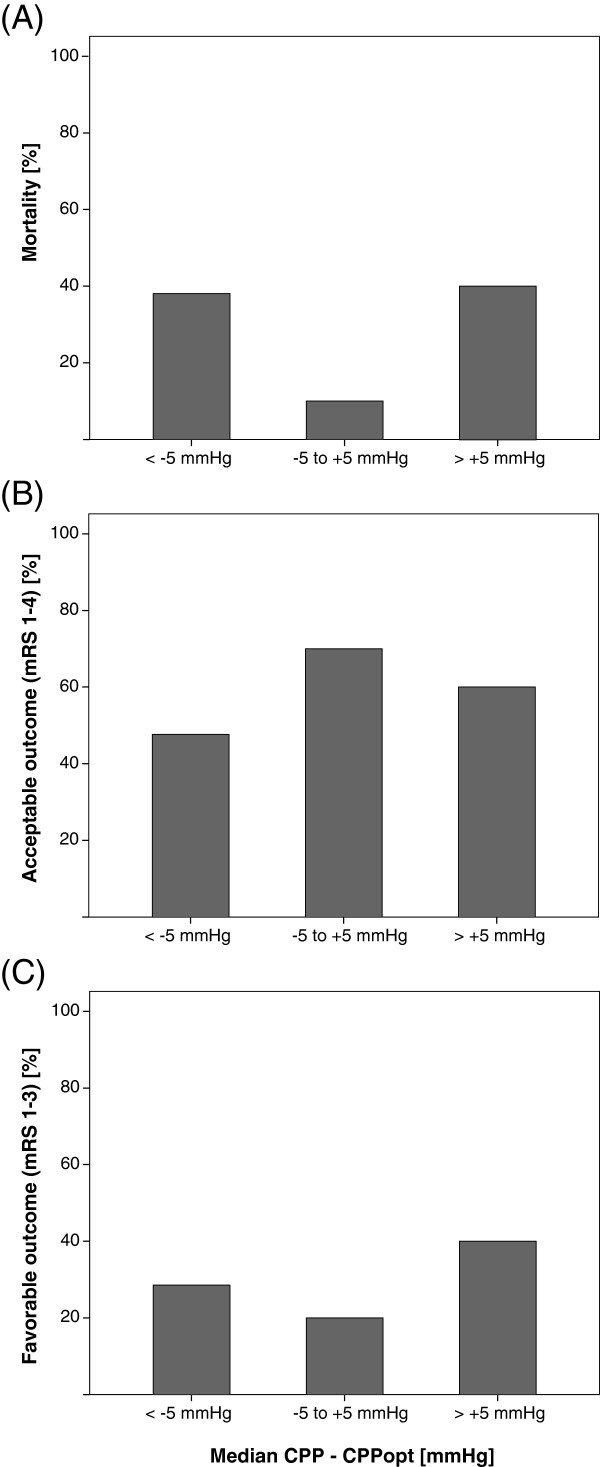
**Relationsship between CPPopt and outcomes.** The relationship between **(A)** mortality, **(B)** acceptable outcome and **(C)** favorable outcome and the individual median difference between continuous calculation of cerebral perfusion pressure (CPP) and optimal CPP using overlapping hourly moving windows of 4 hour duration (n = 36).

The correlation between median Δ and outcome was not assessed since we did not assume a linear relationship. Figure 2 instead depicts the relationship among mortality, acceptable and favorable outcome, and the individual median of the continuously calculated difference between real CPP and CPPopt (mmHg). A total of 36 patients were grouped according to their median Δ (n = 36 since for 1 patient outcome was not available, and in 1 patient CPPopt was not determinable): 21 patients (55.3%) lay 5 mmHg or more below their CPPopt values (median Δ -7.9 mmHg, range -5.5 to -12.9, IQR 4), 10 patients (26.3%) lay ±5 mmHg within their individual CPPopt range (median Δ -1.05 mmHg, range -4.9 to 4.3, IQR 5.6) and 6 patients (15.8%) were 5 mmHg or more above their CPPopt range (median Δ 6.7 mmHg, range 5.2 to 9.4, IQR 2.7).

Mortality was lowest in the group of patients within the CPPopt range (10%), while it was almost equal in those more than 5 mmHg below (38.1%) or above (33.3%) CPPopt (*P* = 0.241, Fisher’s exact test, Figure 2A).

The percentage of patients with acceptable outcome was highest when CPP was within the CPPopt range (70%), compared to 47.6% of those below and 50% of those above CPPopt (*P* = 0.523, Fisher’s exact test, Figure 2B).

In contrast, patients who were more than 5 mmHg above their CPPopt range had the highest percentage of favorable outcome (33.3%), compared to 20% of patients within the CPPopt range and 28.6% of those more than 5 mmHg below CPPopt (*P* = 0.769, Fisher’s exact test, Figure 2C).

The distribution of the NIHSS scores or hemorrhage volume did not differ significantly among the three groups (*P* = 0.630 and *P* = 0.287, Kruskal-Wallis-test).

### Outcome prediction - multivariate models

Mortality at three months was predicted by age and initial hemorrhage volume (Table 1). The only significant predictor of good outcome (mRS 1 to 3) at three months was younger age. Independent predictors for acceptable outcome at three months were lower percentage of time with PRX >0.2 and lower hemorrhage volume. The variable ‘time spent within the CPPopt range’ did not reach significance in any of the models.

**Table 1:** Stepwise logistic regression models to predict outcomes at three months

	**B (SE)**	**OR**	**95% CI**	***P***
**Mortality at three months**^**1**^				
Age	0.08 (0.04)	1.08	1.00 to 1.17	0.042
Hemorrhage volume	0.03 (0.01)	1.03	1.01 to 1.06	0.101
**Good outcome at three months (mRs 1 to 3)**^**2**^				
Age	-0.13 (0.04)	0.88	0.81 to 0.96	0.004
**Acceptable outcome at three months (mRS 1 to 4)**^**3**^				
% time PRx >0.2	-0.06 (0.03)	0.94	0.90 to 1.00	0.038
Hemorrhage volume	−0.02(0.1)	0.98	0.96 to 1.00	0.054

Included variables: age [years], hemorrhage volume [ml], presence of intraventricular hemorrhage, percentage of time with PRx >0.2, percentage of time within the CPPopt range, SAPS II score (^1^Nagelkerkes R^2^ 0.47, ^2^Nagelkerkes R^2^ 0.50, ^3^Nagelkerkes R^2^ 0.38), *B*, regression coefficient; CI, confidence interval; OR, odds ratio; SE, standard error.

## Discussion

To our knowledge, this series is the largest available on dynamic assessment of cerebral autoregulation in severe ICH patients. Furthermore, it is the first study assessing the concept of an autoregulation-oriented CPP management in ICH. We have found that impairment of pressure reactivity occurs in almost half of the cases following spontaneous ICH. The PRx and the time spent with a PRx >0.2 were significantly correlated to mRS at three months. The amount of time a patient had impaired pressure reactivity (PRx >0.2) was an independent predictor for acceptable outcome at three months. These results confirm the findings of a previous pilot study [2]. Importantly, the CPPopt concept seems applicable in ICH patients. In our retrospective, explorative analysis, calculation of CPPopt was feasible in all but one patient and during 57% of the monitoring time. This number is almost identical to the number indicated by Aries *et al*. who reported that identification of CPPopt was on average feasible during 55% of the recording period in their cohort of 326 TBI patients [7]. The median CPPopt value was 83 mmHg and thereby higher than the threshold or 70 mmHg recommended in current guidelines [13,14]. This is potentially interesting since a recent study by Ko *et al*. applying microdialysis and brain tissue oxygenation study in 18 ICH patients showed that CPP values between 50 and 70 mmHg were associated with brain tissue hypoxia, depicting the possibly detrimental effects of hypoperfusion [11]. In our series, however, we did not find a significant relationship between functional outcome at three months and any of the CPPopt variables.

Aries *et al*. have found in their much larger TBI cohort that hypoperfusion was associated with mortality while hyperperfusion was correlated with severe disability. In contrast, in our cohort of ICH patients, both hypo- as well as hyperperfusion seemed harmful as could be concluded from the mortality rates in the different CPP groups. Mortality was lowest for patients close to their individual CPPopt. At the same time, patients in this group had the highest percentage of acceptable outcome. These findings, however, were not statistically significant. In addition, the results for favorable outcome remain inconclusive. In our cohort, the highest percentage of patients with favorable outcome was in the group of those with 5 mmHg or more above their individual CPPopt while at the same time patients with a CPP close to CPPopt had the least percentage of favorable outcomes. This directly contrasts the above mentioned findings with respect to mortality. These inconsistencies may be due to the small sample size and the total lack of patients with a delta CPP above +10 mmHg. Patient numbers in total and especially in the above CPPopt group (n = 6) were too small to draw any definitive conclusions. Furthermore, the variable ‘time spent within the CPPopt’ range was not an independent predictor for any of the outcome variables.

Our study has various important limitations. First, sample size was still relatively small and the study included a highly selected population of ICH patients. As stated above, especially the results of the outcome analyses comparing the different groups based on their proximity to CPPopt did not render any statistically significant results and have to be weighed critically. Only speculative conclusions can be drawn so far. Furthermore, the study was an explorative retrospective analysis of prospectively monitored, non-consecutive patients. Sample size was not predetermined. Second, the amount of available data differed greatly among different individuals, possibly adding further bias. However, reassuringly, total monitoring time was not correlated to outcomes, PRx or time of impaired PRx. Third, CPPopt was calculated retrospectively and the amount of time spent within the CPPopt range was relatively small (11%, range 0 to 33%). In addition, calculation of CPPopt based on the currently used automated algorithm was on average only possible during half of the monitoring time. This may be due to the fact that not all CPP values were covered by each patient, rendering identification of CPPopt impossible since it possibly lay outside the recorded values. This limits the generalizability of the association between the difference of ‘real CPP minus optimal CPP’ and outcome after ICH. However, as stated above, the same percentage (55%) was found in one other retrospective trial using continuous calculation of CPPopt in TBI patients. This number certainly has to be improved by carefully evaluating the reasons for failure of determination of CPPopt and possibilities to increase the numbers. This should be done in a prospective trial. Only after this issue has been addressed can the CPPopt concept be safely introduced for further clinical use. We are aware that a prospectively conducted study, trying to optimize cerebral perfusion based on an improved CPPopt algorithm may render entirely different results due to the availability of larger amounts of data and broader CPP variations. Finally, as a technical limitation, times during CSF drainage as well as patients following surgery and specifically two patients following hemicraniectomy have been included in our analysis. While the NEUROVENT probe allows reliable ICP measurement also during CSF drainage, it is unclear whether this will affect the reliability of PRx assessment. Calculation of PRx is based on the principle of a closed system, with ICP being a surrogate for changes in intracerebral blood volume. However, considering the fact that we clearly found positive correlations between MAP and ICP and hence positive PRx values, we argue that the system still reacts during times of drainage. The same holds true for the two patients following hemicraniectomy. A comparison of PRx values and CPPopt calculation before and after surgery suggests no systematic difference in these two patients (compare Additional file 1: Table S1). If specific patients during CSF drainage would entirely have to be excluded, this would drastically reduce the applicability of PRx for continuous assessment of pressure reactivity in collectives like ICH or SAH. A study on this issue is thus far missing. However, there are data indicating that in situations with high brain compliance, for example, following surgery, the PRx may not be an ideal parameter, suggesting a different index PAx as an alternative, especially in situations with lower ICP [17]. Furthermore, a non-invasive alternative for continuous determination of cerebrovascular reactivity and CPPopt may be the near-infrared spectroscopy based index THx. The index has been evaluated in TBI patients [18]. However, the applicability of THx in the setting of high brain compliance has not been addressed. Furthermore, the correlation between the autoregulation indices varies around 0.5 [18] and 0.6 [17], indicating that different techniques may represent distinct aspects of cerebrovascular reactivity and autoregulation. So far, the PRx remains the best studied parameter, especially with respect to calculation of CPPopt and probably would be the parameter of choice in a prospective trial on CPPopt management.

Despite these important limitations, the study encourages further research on autoregulation-based CPP management in ICH. Moreover, our finding of impaired dynamic autoregulation following ICH is corroborated by two other independent studies using the Doppler flow velocity-based index Mx [3,9]; one of them also reports a relationship between impaired autoregulation and worse functional outcome [3]. Further indirect arguments in favor of individualized thresholds have just been provided in a recent study on ICP management in TBI patients [19]. Chestnut and colleagues have compared an ICP-based treatment protocol to a protocol solely based on clinical observations. The outcomes in both groups did not differ. The authors stress that their findings do not argue against the use of ICP monitoring in general but only argue against the monitoring-based interventional algorithm of lowering ICP >20 mmHg. They suggest that the lack of efficacy of the ICP-based protocol may be attributable to the use of a universal threshold for intracranial pressure. Currently, a protocol for a prospective multicenter trial comparing standard CPP management versus autoregulation-based CPP management is being designed.

## Conclusions

Due to a lack of ICH specific data, current guidelines for CPP management in ICH recommend taking the functioning of cerebral autoregulation into account but are entirely based on data from TBI patients. Here we could show that cerebrovascular pressure reactivity is impaired in almost 50% of patients with severe ICH and impairment of pressure reactivity was related to poor functional outcome at three months. The CPPopt concept seems applicable to ICH patients: CPPopt could be determined in all but one patient and during 57% of the monitoring time. The median CPPopt was 83 mmHg and, therefore, considerably higher than the cutoff value of 70 mmHg recommended in the current guidelines. So far, however, a significant relationship between functional outcome and CPPopt could not be demonstrated in ICH patients. This may be due to sample size and the retrospective approach. Larger, prospective trials are needed to assess the validity of the CPPopt concept for severe ICH patients.

## Key messages

● Cerebrovascular pressure autoregulation was impaired in half of the patients treated for severe ICH.

● Impairment of pressure reactivity is independently associated with poor functional outcome at three months.

● The CPPopt concept seems applicable in ICH: CPPopt was determinable in 57% of the total monitoring time.

● The median CPPopt was 83 mmHg and thereby considerably higher than the guideline recommended value of 70 mmHg.

● Mortality was lowest in the group of patients with a CPP close to their individual CPPopt.

## Abbreviations

B: Regression coefficient; CI: Confidence interval; CPP: Cerebral perfusion pressure; CPPopt: Optimal cerebral perfusion pressure; CSF: Cerebrospinal fluid; CT: Computed tomography; EVD: External ventricular drain; ICH: Intracerebral haemorrhage; ICP: Intracranial pressure; IQR: Interquartile range; MAP: Mean arterial pressure; mRS: Modified Rankin Scale; NCCU: Neurocritical Care Unit; NIHSS: National Institutes of Health Stroke Scale; OR: Odds ratio; PaCO2: Arterial pressure of carbon dioxide; PRx: Pressure reactivity index; R: Correlation coefficient; SAH: Subarachnoid hemorrhage; SAPS II: Simplified Acute Physiology Score II; SE: Standard error; TBI: Traumatic brain injury; Δ: Delta.

## Competing interests

The authors declare that they have no competing interests.

## Authors’ contributions

JD contributed to conception and design, data collection and analysis, manuscript writing and final approval of the manuscript. ES and SP contributed to data collection and analysis, critical revision and final approval of the manuscript. MS contributed to conception and design, data collection and analysis, critical revision and final approval of the manuscript. All authors read and approved the final manuscript.

## Supplementary Material

Additional file 1: Table S1 Comparison of PRx values and CPPopt assessment before and after surgery for the two patients who had hemicraniectomy. Click here for file
