# Supplementary material for: Optimal cerebral perfusion pressure in patients with intracerebral hemorrhage: an observational case series
Source: Crit Care. 2014 Mar 25;18(2):R51. doi: 10.1186/cc13796 (PMC4057227; doi:10.1186/cc13796)
Supplement: Additional file 1: Table S1 — Comparison of PRx values and CPPopt assessment before and after surgery for the two patients who had hemicraniectomy. [file cc13796-S1.docx]

| Pat #1 | PRx | monitoring time (MT) [h] | CPPopt calculable [% MT] | CPPopt |
| --- | --- | --- | --- | --- |
| before surgery | 0.24 | 47 | 48.0% | 87.5 |
| after surgery | 0.25 | 63 | 45.7% | 77.5 |
| Pat #2 |  |  |  |  |
| before surgery | -0.17 | 21 | 57.1% | 87.5 |
| after surgery | 0.04 | 71 | 78.1% | 87.5 |

Supplementary table 1: Comparison of PRx values and CPPopt assessment before and after surgery for the two patients who had hemicraniectomy.
